# Fetal Reduction Could Improve but Not Completely Reverse the Pregnancy Outcomes of Multiple Pregnancies: Experience From a Single Center

**DOI:** 10.3389/fendo.2022.851167

**Published:** 2022-06-24

**Authors:** Zhu Yimin, Tang Minyue, Fu Yanling, Yan Huanmiao, Sun Saijun, Li Qingfang, Hu Xiaoling, Xing Lanfeng

**Affiliations:** Department of Reproductive Endocrinology, Women’s Hospital, Zhejiang University School of Medicine, Hangzhou, China

**Keywords:** multifetal pregnancy reduction, assisted reproductive technology, pregnancy outcome, twins, triplets

## Abstract

**Objective:**

To investigate the effectiveness and limitations of multifetal pregnancy reduction (MFPR) on the improvement of pregnancy outcomes of triplet or twin pregnancies conceived by *in vitro* fertilization (IVF) or intracytoplasmic sperm injection (ICSI).

**Methods:**

We performed a cohort study of women undergoing IVF or ICSI from 2002–2016 in reproductive center, women’s hospital, Zhejiang University School of Medicine. The cohort included 502 women who underwent MFPR and 9641 non-reduced women. Pregnancy outcomes were gestational age (GA) at delivery, pregnancy loss, preterm delivery, low birth weight (LBW), very low birth weight (VLBW), and small for gestational age (SGA). Multiple linear regression and logistic regression models were used to compare pregnancy outcomes between groups.

**Results:**

Triplets reduced to singletons had a longer median GA (39.07 vs 37.00, P<0.001), and lower rates of LBW (8.9% vs 53.2%, P<0.001) and SGA (17.8% vs 44.7%, P=0.001) than triplets reduced to twins, with a similar pregnancy loss rate (6.7% vs 6.6%, P=0.701). Twins reduced to singletons had a comparable pregnancy loss rate (4.8% vs. 6.5%, P=0.40), a longer median GA (38.79 vs. 37.00, P<0.001), and lower rates of LBW (13.5% vs. 47.0%, P<0.001) and SGA (13.5% vs. 39.6%, P<0.001) than primary twins. Triplets reduced to twins had higher rates of LBW (53.2% vs. 47.0%, P=0.028) and SGA (44.7% vs. 39.6%, P=0.040) than primary twins, with a similar pregnancy loss rate (6.6% vs. 6.5%, P=0.877). Singletons reduced from triplets/twins had higher rates of preterm delivery (15.8% vs. 7.3%, P<0.001), LBW (12.3% vs. 4.32%, P<0.001), VLBW (2.3% vs. 0.4%, P=0.002), and SGA (14.6% vs.6.6%, P<0.001) than primary singletons, with a comparable pregnancy loss rate (5.3% vs. 5.4%, P=0.671).

**Conclusions:**

This study suggests that the pregnancy loss rate is similar between reduction and non-reduction groups. MFPR improves pregnancy outcomes, including the risk of preterm delivery, LBW, and SGA, but still could not completely reverse the adverse pregnancy outcomes of multiple pregnancies.

## Introduction

There has been a growing trend for the increasing use of assisted reproductive technology (ART) to combat infertility in recent years. However, ART constitutes a major risk factor for the prevalence of multiple pregnancies (1, 2). Multiple pregnancies are associated with an increasing risk for mothers and fetuses, including maternal complications, as well as low birth weight (LBW) and small for gestational age (SGA) (3, 4).

As the risks of multiple pregnancies have gradually been recognized, several countries have legally mandated a decrease in the number of embryos transferred and advocated for elective single embryo transfer (SET) (5–8). However, transfer of more than one embryo is still common in many countries (9, 10). Multifetal pregnancy reduction (MFPR) is a secondary preventive measure for managing multiple pregnancies that have occurred. MFPR began in the 1980s to salvage pregnancies with too many fetuses by ART (11). Because MFPR is an interventional operation, a major difficulty is the lack of clarity regarding the explicit benefits and limitations of MFPR when counselling for triplet or twin pregnancies conceived by IVF or ICSI (12). It can always be difficult for couples with triplet or twin pregnancies conceived by IVF or ICSI to weigh the pros and cons to decide whether to reduce fetus since the fetuses are hard-won for them. In recent years, with a growing awareness of the adverse outcomes of multiple pregnancies and accumulating data supporting the safety of MFPR, reduction of triplets is a widely accepted option (13, 14). However, for triplet pregnancies, reducing to singles or twins is still a tough decision. Moreover, the effectiveness of reduction from twins to singletons is controversial (15–17). Previous studies regarding pregnancy outcomes after MFPR were based on limited and conflicting data, which require further investigation.

In this study, we aimed to address this inconsistency and to further investigate whether MFPR get equal benefit as primary singleton/twin pregnancies using a large dataset from a single center during 15 years. Pregnancy outcomes of triplets and twins who underwent MFPR were recorded, and the benefits and limitations from reduction were evaluated to provide a comprehensive understanding of MFPR.

## Materials and Methods

### Study Design

**Figure 1:**
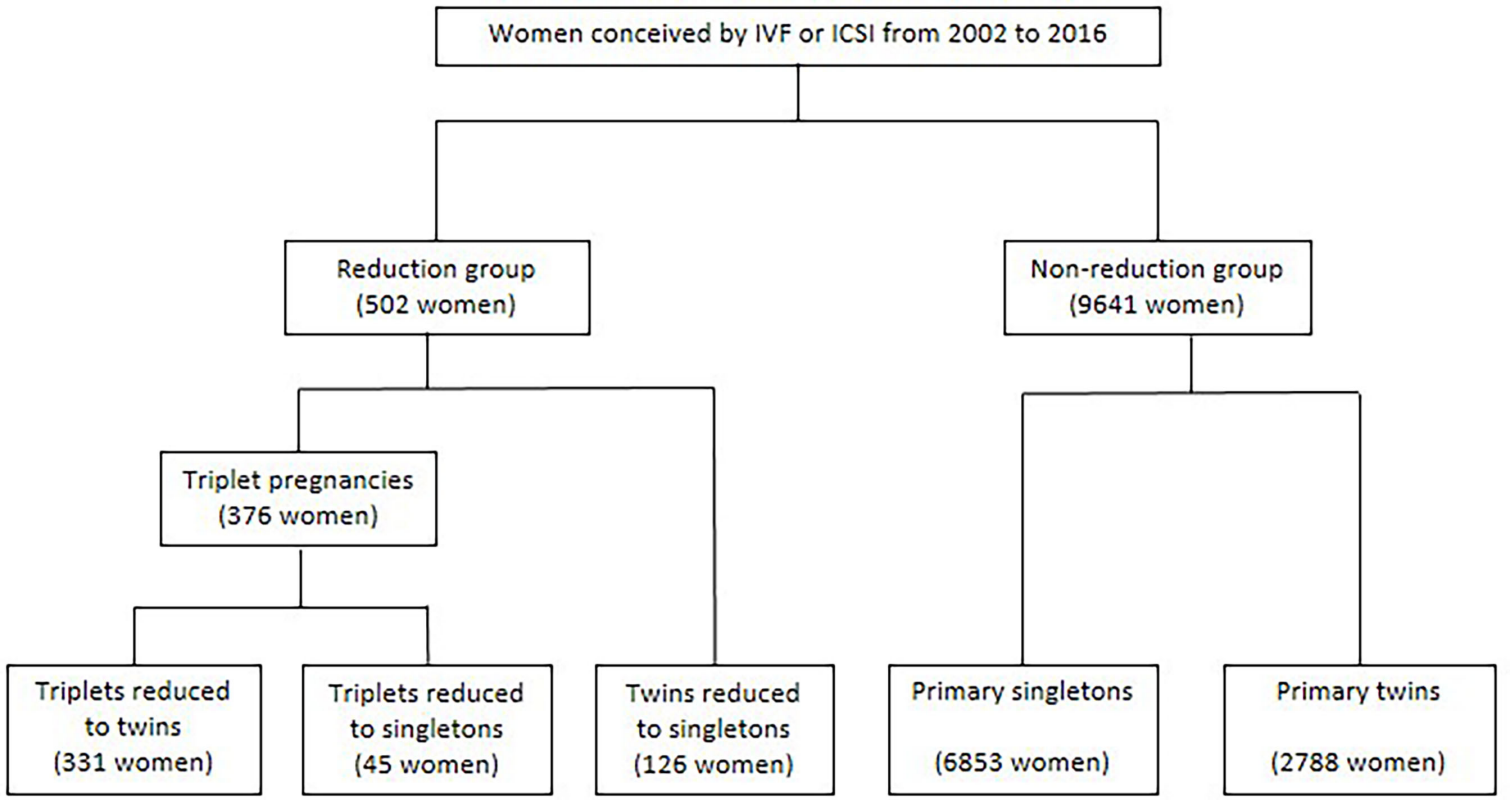
Study design.

This is a cohort study performed in reproductive center, women’s hospital, Zhejiang University School of Medicine (**Figure 1**). The reduction group included a cohort of multiple-pregnant women conceived by IVF or ICSI who underwent MFPR and continued follow-up in the reproductive center from 2002 to 2016. Exclusion criteria for the reduction group: 1) initial fetuses >3;2) ectopic pregnancy;3) heterotopic pregnancy; 4) ART or reduction data missed in database; 5) data on pregnancy outcomes were not available. After exclusion, the reduction group included a cohort of 502 women conceived after ART with triplet or twin pregnancies and reduced to twins or singletons at 6-16 weeks of gestation. In this cohort, there were 331 women with twins reduced from triplets at 6-13 weeks, 45 women with singletons reduced from triplets at 7-12 weeks, 126 women with singletons reduced from twins at 7-16 weeks. This study was approved by the Women’s hospital, Zhejiang University School of Medicine, and written informed consents were obtained from all participants.

All women conceived by IVF or ICSI at our center during the same period without undergoing MFPR who met the inclusion criteria were enrolled in the non-reduction group. Inclusion criteria for non-reduction group: 1) with integrated ART data in database; 2) underwent ultrasound exam at our center confirmed singletons or twins intrauterine live pregnancies between 6-8 weeks of gestation; 3) continued follow-up in this reproductive center from 2002 to 2016. The non-reduction group included 2788 women with primary twins and 6853 women with primary singletons.

All MFPR procedures were carried out by highly skilled physicians. All patients underwent counseling regarding the risks and benefits of MFPR, and were advised to reduce the number of embryos to one or two, depending on previous obstetric history, religious beliefs, and patient preference. The reason for reduction could be either a genetic abnormality or structural abnormality in one or two of the fetuses diagnosed by ultrasound or an invasive diagnostic test, the prevention of preterm birth or completely elective. Fetal reduction procedures were performed transvaginal between 6 to 16 weeks gestation. The patient with an empty bladder was in the lithotomy position. After cleaning the vagina with povidone iodine, the fetuses were visualized using a transvaginal ultrasound transducer to verify the number, position, size, and heart activity of each fetuses. The smallest or abnormal fetuses and/or the fetuses that was located in a position with the easiest access route was selected for reduction. An appropriate size needle was inserted into the fetal heart to aspirate the fluid and fetus from the sac or inject potassium chloride solution. Prophylactic antibiotic therapy was used for 3 days in all cases. Women were discharged from the clinic after bed rest and an average observation period of 120 minutes. A follow-up ultrasound was carried out within 1 week.

Maternal and ART characteristics were prospectively recorded in inpatient database of reproductive center, women’s hospital, Zhejiang University. All patients underwent subsequent follow-up by telephone. Delivery and offspring characteristics were collected through telephone interview and review of medical records. GA was calculated based on the embryo transfer (ET) time and was correlated to a first trimester ultrasound exam. Reduction weeks was defined as the GA at the MFPR. Parental characteristics included maternal age at conception, height, weight and BMI before pregnancy. ART characteristics included type of infertility, ART methods, embryo transplantation, and source of semen. Primary infertility was defined as the inability to ever become pregnant after at least one year of having sex and not using birth control methods. Secondary infertility was defined by the inability of a couple that already has conceived and delivered a newborn to conceive again. ART methods were defined as IVF or ICSI, ET was defined as fresh-ET or Frozen-ET, source of semen was defined as ejaculated semen, sperm aspiration and donor semen.

### Outcomes

Pregnancy outcomes assessed in this study included GA at delivery, the rates of preterm delivery before <32 weeks, <34 weeks, and <37 weeks of gestation, pregnancy loss < 24 weeks, abortion of one fetus and caesarean section as well as neonatal outcomes such as neonatal birth weight, the rates of at least one fetus LBW, at least one fetus very low birth weight (VLBW) and SGA. LBW was defined as birth weight below 2500g, and VLBW was defined as birth weight below 1500g. SGA was defined as birth weight below the 10th percentile for the gestational age at delivery (18).

### Statistical Analysis

Comparison of continuous variables was analyzed using Mann–Whitney U tests. Categorical variables were compared by using Chi-square tests or Fisher’s exact test. Logistic regression and linear regression were used for adjusting certain confounders. Significance was accepted at P<0.05. All reported P values were two-sided. Statistical analyses were conducted using the IBM SPSS 23.0 (IBM, USA).

## Results

The study cohort and groups are shown in **Figure 1**. The demographics and ART characteristics for five groups are given in **Table 1**.

**Table 1:** Demographic and ART characteristics.

	Primary singletons(n=6853)	Primary twins(n=2788)	Twins reduced to singletons (n=126)	Triplets reduced to singletons (n=45)	Triplets reduced to twins (n=331)	*P* value 1	*P* value 2	*P* value 3	*P* value 4	*P* value 5
**Maternal age at conception**	30 (28-33)	30 (28-33)	33 (29-35)	30 (28-35)	31 (28-34)	<0.001	0.616	0.474	<0.001	<0.001
**Maternal height before pregnancy**	160.0 (157.0-163.0)	160.0 (157.0-163.0)	159.0 (155.0-162.3)	158.0 (156.0-160.0)	160.0 (157.0-163.0)	0.786	0.123	0.118	0.056	0.073
**Maternal weight before pregnancy**	55.0 (50.4-60.0)	55.0 (51.0-61.0)	56.0 (50.9-60.1)	53.0 (48.0-58.8)	55.0 (51.0-60.0)	0.426	0.090	0.087	0.778	0.558
**Maternal BMI before pregnancy**	21.6 (20.0-23.6)	21.7 (20.0-23.9)	22.2 (20.3-23.9)	20.9 (19.5-23.4)	21.6 (20.0-23.7)	0.407	0.242	0.203	0.346	0.070
**Type of infertility**						0.005	0.004	0.070	0.026	0.048
Primary infertility	3054 (44.6)	1278 (45.8)	45 (35.7)	14 (31.1)	179 (54.1)					
Secondary infertility	3799 (55.4)	1510 (54.2)	81 (64.3)	31 (68.9)	152 (45.9)					
**ART methods**						<0.001	0.073	0.435	0.125	0.212
IVF	5774 (84.3)	2374 (85.2)	101 (80.2)	36 (80.0)	221 (66.8)					
ICSI	1079 (15.7)	414 (14.8)	25 (19.8)	9 (20.0)	110 (33.2)					
**Embryo transplantation**						0.070	0.094	0.934	0.134	0.775
Fresh-ET	3460 (50.5)	1561 (56.0)	62 (49.2)	23 (51.1)	168 (50.8)					
Frozen-ET	3393 (49.5)	1227 (44.0)	64 (50.8)	22 (48.9)	163 (49.2)					
**Source of semen**						0.731	0.178	0.019	0.191	0.254
Ejaculated semen	6623 (96.6)	2668 (95.7)	125 (99.2)	41 (91.1)	316 (95.5)					
Sperm aspiration	154 (2.2)	76 (2.7)	1 (0.8)	1 (2.2)	8 (2.4)					
Donor Semen	76 (1.1)	44 (1.6)	0	3 (6.7)	7 (2.1)					
**Reduction weeks**	–	–	8.3 (7.7-9.0)	8.1 (7.7-8.6)	8.0 (7.7-8.4)	–	0.161	–	–	–

Data are presented as median (IQR) or number (%). Comparison of continuous variables was analyzed using Mann–Whitney U tests. Categorical variables were compared by using Chi-square tests or Fisher’s exact test. P value 1 represents for triplets reduced to twins versus primary twins; P value 2 represents for triplets reduced to twins versus triplets reduced to singleton; P value 3 represents for triplets reduced to singleton versus primary singleton; P value 4 represents for twins reduced to singleton versus primary twins; P value 5 represents for twins reduced to singleton versus primary singleton;

BMI, body mass index (kg/m^2^); ART, assisted reproduction technique; IVF, in vitro fertilization; ICSI, intracytoplasmic sperm injection; ET, embryo transplantation; TESA, testicular sperm aspiration; PESA, percutaneous epididymal sperm aspiration; MESA, microsurgical epididymal sperm aspiration. Sperm aspiration includes TESA, PESA, and MESA.

### Triplets Reduced to Singletons Versus Triplets Reduced to Twins

Pregnancy outcomes of the two groups are shown in **Table 2**. For triplets reduced to singletons, the median GA at delivery was more than 2 weeks longer than that for triplets reduced to twins (39.07 vs 37.00 weeks; P<0.001). Triplets reduced to singletons had significantly lower rates of preterm delivery at <37 weeks (13.3% vs 45.3%; adjusted OR, 0.58; 95% CI, 0.43–0.79; P<0.001) and cesarean section compared with triplets reduced to twins (66.7% vs 93.9%; adjusted OR, 0.51; 95% CI, 0.38–0.67; P<0.001). There was no difference observed in rate of preterm delivery at <32 weeks (2.2% vs 5.1%, P=0.412) or <34 weeks (4.4% vs 11.5%, P=0.206). Similarly, no significant differences were found in the rates of pregnancy loss at <24 weeks (6.7% vs 6.6%) and at least one VLBW (0% vs 3.3%) between the two groups. Singletons reduced from triplets had a significantly higher rate of all surviving (93.3% vs 79.5%, adjusted OR, 1.62; 95% CI, 1.07-2.44; P=0.023), and higher median birth weight than twins reduced from triplets (3050 g vs 2500 g, P <0.001, **Figure S1**). Women with singletons reduced from triplets had a significantly lower risk of having at least one LBW neonate compared with women with twins reduced from triplets (8.9% vs 53.2%; adjusted OR, 0.44; 95% CI, 0.31–0.63; P<0.001). Additionally, we analyzed the incidence of SGA to exclude the effect of different gestational ages. Women with singletons reduced from triplets had a significantly lower risk of having at least one SGA neonate than women with twins reduced from triplets (17.8% vs 44.7%; adjusted OR, 0.62; relative risk, 0.48–0.82, P=0.001).

**Table 2:** Pregnancy outcomes of triplets reduced to singletons versus triplets reduced to twins.

	Triplets reduced to singleton(n=45)	Triplets reduced to twins(n=331)	Unadjusted *P* Value	Unadjusted OR(95%CI)	Adjusted *P* Value	Adjusted OR(95% CI)
**GA at delivery**	39.07 (38.25-40.04)	37.00 (35.71-37.86)	<0.001	–	<0.001	–
**Delivery<32weeks**	1 (2.2)	17 (5.1)	0.626	0.42 (0.06-3.23)	0.412	0.75 (0.37-1.50)
**Delivery<34weeks**	2 (4.4)	38 (11.5)	0.239	0.36 (0.08-1.54)	0.206	0.73 (0.44-1.19)
**Delivery<37weeks**	6 (13.3)	150 (45.3)	<0.001	0.19 (0.08-0.45)	<0.001	0.58 (0.43-0.79)
**Pregnancy loss <24weeks**	3 (6.7)	22 (6.6)	1.000	1.00 (0.19-3.50)	0.701	0.92 (0.59-1.42)
**All surviving**	42 (93.3)	263 (79.5)	0.026	3.62 (1.09-12.03)	0.023	1.62 (1.07-2.44)
**Caesarean section**	28/42 (66.7)	290/309 (93.9)	<0.001	0.13 (0.06-0.29)	<0.001	0.51 (0.38-0.67)
**Birth weight (g)**	3050 (2775-3300)	2500 (2200-2800)	<0.001	–	<0.001	–
**At least one LBW**	4 (8.9)	176 (53.2)	<0.001	0.09 (0.03-0.25)	<0.001	0.44 (0.31-0.63)
**At least one VLBW**	0	11 (3.3)	0.441	–	0.997	–
**At least one SGA**	8 (17.8)	148 (44.7)	0.001	0.27 (0.12-0.59)	0.001	0.62 (0.48-0.82)

Data are presented as median (IQR) or number (%). Mann–Whitney U tests and Chi-square tests or Fisher’s exact test were used for unadjusted analysis. Logistic regression and linear regression were used for adjusting certain confounders, including maternal age at conception, maternal BMI before pregnancy, type of infertility, ART methods, embryo transplantation, source of semen, and weeks of reduction.

GA, gestational age; LBW, low birth weight; VLBW, very low birth weight; SGA, small for gestational age; OR, odds ratio; CI, confidence interval.

### Twins Reduced to Singletons Versus Primary Twins

Pregnancy outcomes of twins reduced to singletons and primary twins are shown in **Table 3**. No significant differences were found in the rates of pregnancy loss at <24 weeks (4.8% vs 6.5%), preterm delivery at <32 weeks (4.0% vs 4.5%) or <34 weeks (5.6% vs 10.1%), and at least one VLBW (3.2% vs 3.8%) between the two groups. The rate of all surviving was significantly higher in twins reduced to singletons than primary twins (78.5% vs 95.2%; adjusted OR, 1,44; 95% CI, 1.22-1.70; P<0.001). For twins reduced to singletons, the median GA at delivery was 38.79 weeks, which was significantly longer than 37.00 weeks for primary twins (P<0.001). Twins reduced to singletons had significantly lower rates of preterm delivery at <37 weeks (16.7% vs 44.5%; adjusted OR, 0.76; 95% CI, 0.69–0.83; P<0.001) and cesarean section (76.7% vs 92.7%; adjusted OR, 0.76; 95% CI, 0.69–0.83; P<0.001) compared with primary twins. Singletons reduced from twins had a significantly higher birth weight (3080g vs 2550 g, P<0.001, **Figure S1**) and significantly lower rate of at least one LBW (13.5% vs 47.0%, adjusted OR, 0.71; 95% CI, 0.64-0.79; P<0.001) compared with primary twins. The incidence of at least one SGA in singletons reduced from twins was significantly lower than that in primary twins (13.5% vs 39.6%; adjusted OR, 0.75; 95% CI, 0.68–0.85; P<0.001).

**Table 3:** Pregnancy outcomes of twins reduced to singletons versus primary twins.

	Primary twins(n=2788)	Twins reduced to singleton(n=126)	Unadjusted *P* Value	Unadjusted OR(95% CI)	Adjusted *P* Value	Adjusted OR(95% CI)
**GA at delivery**	37.00 (35.71-38.00)	38.79 (37.46-39.43)	<0.001	–	<0.001	–
**Delivery<32weeks**	126 (4.5)	5 (4.0)	0.770	0.87 (0.35-2.17)	0.743	0.97 (0.81-1.17)
**Delivery<34weeks**	281 (10.1)	7 (5.6)	0.096	0.53 (0.24-1.14)	0.116	0.88 (0.76-1.03)
**Delivery<37weeks**	1240 (44.5)	21 (16.7)	<0.001	0.25 (0.16-0.40)	<0.001	0.76 (0.69-0.83)
**Pregnancy loss <24weeks**	182 (6.5)	6 (4.8)	0.430	0.72 (0.31-1.65)	0.400	0.93 (0.79-1.10)
**All surviving**	2188(78.5)	120 (95.2)	<0.001	5.48 (2.40-12.52)	<0.001	1.44(1.22-1.70)
**Caesarean section**	2401/2591 (92.7)	92/120 (76.7)	<0.001	0.26 (0.17-0.41)	<0.001	0.76 (0.69-0.83)
**Birth weight (g)**	2550 (2225-2850)	3080 (2750-3350)	<0.001	–	<0.001	–
**At least one LBW**	1311 (47.0)	17 (13.5)	<0.001	0.18 (0.11-0.30)	<0.001	0.71 (0.64-0.79)
**At least one VLBW**	106 (3.8)	4 (3.2)	0.903	0.83 (0.30-2.29)	0.703	0.96 (0.78-1.18)
**At least one SGA**	1103 (39.6)	17 (13.5)	<0.001	0.24 (0.14-0.40)	<0.001	0.75 (0.68-0.85)

Data are presented as median (IQR) or number (%). Mann–Whitney U tests and Chi-square tests or Fisher’s exact test were used for unadjusted analysis. Logistic regression and linear regression were used for adjusting certain confounders, including maternal age at conception, maternal BMI before pregnancy, type of infertility, ART methods, embryo transplantation, source of semen.

GA, gestational age; LBW, low birth weight; VLBW, very low birth weight; SGA, small for gestational age; OR, odds ratio; CI, confidence interval.

### Triplets Reduced to Twins Versus Primary Twins

Pregnancy outcomes of triplets reduced to twins versus primary twins are given in **Table 4**. No significant differences were found in the rates of preterm delivery at <32 weeks (5.1% vs 4.5%), <34 weeks (11.5% vs 10.5%), and <37 weeks (45.3% vs 44.5%), pregnancy loss at <24 weeks (6.6% vs 6.5%), abortion of one fetus (13.9% vs 14.1%), all surviving (78.5% vs 79.5%), cesarean section (93.9% vs 92.7%) between twins reduced from triplets and primary twins. Likewise, median birth weight (2500 vs 2550 g, P=0.195, **Figure S1**) and the rate of at least one VLBW (3.3% vs 3.8%, P=0.708) were also comparable between two groups. Twins reduced from triplets had a significantly higher rate of at least one LBW (53.2% vs 47.0%; adjusted OR, 1.07; 95% CI, 1.01–1.13; P=0.028) compared with primary twins. Additionally, the incidence of at least one SGA in triplets reduced to twins was significantly higher than that in primary twins (44.7% vs 39.6%; adjusted OR, 1.06; 95% CI, 1.00–1.13; P=0.040).

**Table 4:** Pregnancy outcomes of triplets reduced to twins versus primary twins.

	Primary twins(n=2788)	Triplets reduced to twins(n=331)	Unadjusted *P* Value	Unadjusted OR (95% CI)	Adjusted *P* Value	Adjusted OR (95% CI)
**GA at delivery**	37.00 (35.71-38.00)	37.00 (35.71-37.86)	0.992	–	0.957	–
**Delivery<32weeks**	126 (4.5)	17 (5.1)	0.612	1.14 (0.68-1.92)	0.590	1.04 (0.91-1.19)
**Delivery<34weeks**	281 (10.1)	38 (11.5)	0.426	1.16 (0.81-1.66)	0.284	1.05 (0.96-1.15)
**Delivery<37weeks**	1240 (44.5)	150 (45.3)	0.771	1.04 (0.82-1.30)	0.764	1.01 (0.95-1.07)
**Pregnancy loss <24weeks**	182 (6.5)	22 (6.6)	0.934	1.02 (0.65-1.61)	0.877	1.01 (0.90-1.14)
**Abortion of one fetus**	392 (14.1)	46 (13.9)	0.936	0.99 (0.71-1.37)	0.549	0.98 (0.90-1.06)
**All surviving**	2188(78.5)	263(79.5)	0.682	1.06 (0.80-1.41)	0.397	1.03(0.96-1.11)
**Caesarean section**	2401/2591 (92.7)	290/309 (93.9)	0.447	1.21 (0.74-1.97)	0.371	1.06 (0.94-1.20)
**Birth weight (g)**	2550 (2225-2850)	2500 (2200-2800)	0.130	–	0.195	–
**At least one LBW**	1311 (47.0)	176 (53.2)	0.034	1.28 (1.02-1.61)	0.028	1.07 (1.01-1.13)
**At least one VLBW**	106 (3.8)	11 (3.3)	0.665	0.87 (0.46-1.64)	0.708	0.97 (0.83-1.14)
**At least one SGA**	1103 (39.6)	148 (44.7)	0.071	1.24 (0.98-1.55)	0.040	1.06 (1.00-1.13)

Data are presented as median (IQR) or number (%). Mann–Whitney U tests and Chi-square tests or Fisher’s exact test were used for unadjusted analysis. Logistic regression and linear regression were used for adjusting certain confounders, including maternal age at conception, maternal BMI before pregnancy, type of infertility, ART methods, embryo transplantation, source of semen.

Birth weight, at least one LBW, and at least one VLBW were additionally adjusted for GA at delivery.

GA, gestational age; LBW, low birth weight; VLBW, very low birth weight; SGA, small for gestational age; OR, odds ratio; CI, confidence interval.

### Triplets/Twins Reduced to Singletons Versus Primary Singletons

Pregnancy outcomes of triplets/twins reduced to singletons and primary singletons are shown in **Table 5**. Triplet/twin pregnancies reduced to singletons included 126 singletons reduced from twins and 45 singletons reduced from triplets. No significant differences were found in the rates of pregnancy loss at <24 weeks (5.4% vs 5.3%) and live birth (94.1% vs 94.7%) between the groups. Although GA at delivery was comparable between the two groups, analysis across different GA cut–offs showed a significant disadvantage for triplet/twin pregnancies reduced to singletons, with higher rates of preterm delivery either at <37 weeks (15.8% vs 7.3%; adjusted OR, 1.51; 95% CI, 1.07–1.24; P<0.001), <34 weeks (5.3% vs 1.7%; adjusted OR, 1.22; 95% CI 1.09–1.37; P=0.001), or <32 weeks (3.5% vs 0.9%; adjusted OR, 1.25; 95% CI, 1.08–1.44; P=0.002). Newborns in triplet/twin pregnancies reduced to singletons had significantly lower median birth weights (3055 vs 3340 g, P<0.001, **Figure S1**) and higher rates of LBW (12.3% vs 4.3%; adjusted OR, 1.21; 95% CI, 1.11–1.30; P<0.001) and VLBW (2.3% vs 0.4%; adjusted OR, 1.32; 95% CI, 1.10–1.58; P=0.002) compared with primary singletons. Additionally, the incidence of SGA in triplets/twins reduced to singletons was significantly higher than that in primary twins (14.6% vs 6.6%; adjusted OR, 1.17; 95% CI, 1.09–1.26; P<0.001). Additionally, the comparison between triplets reduced to singletons and primary singletons, twins reduced to singletons and primary singletons are given in **Table S1**.

**Table 5:** Pregnancy outcomes of triplets or twins reduced to singletons versus primary singletons.

	Primary singletons(n=6853)	Triplets/twins reduced to singletons (n=171)	Unadjusted *P* Value	Unadjusted OR (95%CI)	Adjusted *P* Value	Adjusted OR (95%CI)
**GA at delivery**	39.00 (38.0-40.0)	38.93 (37.71-39.57)	0.830	–	0.155	–
**Delivery<32weeks**	65 (0.9)	6 (3.5)	0.004	3.80 (1.62-8.89)	0.002	1.25 (1.08-1.44)
**Delivery<34weeks**	119 (1.7)	9 (5.3)	0.002	3.14 (1.57-6.30)	0.001	1.22 (1.09-1.37)
**Delivery<37weeks**	500 (7.3)	27 (15.8)	<0.001	2.38 (1.56-3.63)	<0.001	1.51 (1.07-1.24)
**Pregnancy loss <24weeks**	371 (5.4)	9 (5.3)	0.932	0.97 (0.49-1.92)	0.671	0.98 (0.87-1.09)
**Live birth**	6450 (94.1)	162 (94.7)	0.734	1.13 (0.57-2.22)	0.496	1.04 (0.93-1.17)
**Caesarean section**	4871/6463 (75.4)	120/162 (74.1)	0.706	0.93 (0.65-1.33)	0.325	0.97 (0.91-1.03)
**Birth weight (g)**	3340 (3038.73-3650)	3055 (2750-3312.50)	<0.001	–	<0.001	–
**LBW**	295 (4.3)	21 (12.3)	<0.001	3.11 (1.94-4.99)	<0.001	1.21 (1.11-1.30)
**VLBW**	29 (0.4)	4 (2.3)	0.008	5.64 (1.96-16.21)	0.002	1.32 (1.10-1.58)
**SGA**	451 (6.6)	25 (14.6)	<0.001	2.43 (1.57-3.76)	<0.001	1.17 (1.09-1.26)

Data are presented as median (IQR) or number (%). Mann–Whitney U tests and Chi-square tests or Fisher’s exact test were used for unadjusted analysis. Logistic regression and linear regression were used for adjusting certain confounders, including maternal age at conception, maternal BMI before pregnancy, type of infertility, ART methods, embryo transplantation, source of semen.

GA, gestational age; LBW, low birth weight; VLBW, very low birth weight; SGA, small for gestational age; OR, odds ratio; CI, confidence interval.

## Discussion

This cohort study showed that MFPR improved pregnancy outcomes, including preterm delivery, LBW, and SGA, but still could not completely reverse the adverse pregnancy outcomes of multiple pregnancies. Additionally, MFPR was a relatively safe operation that did not increase pregnancy loss at <24 weeks. To the best of our knowledge, this is the largest study to compare the pregnancy outcomes of transvaginal MFPR in women with triplet or twin pregnancies, which provides a systematic and comprehensive interpretation to the benefits and limitations of MFPR.

Multiple pregnancies are an inevitable consequence of more than one embryo transfer in ART, which is responsible for increasing risks in prematurity (19). Numerous studies have shown that twins reduced from triplets have better pregnancy outcomes than ongoing triplets (13, 14, 20). Therefore, the benefits of MFPR for triplet pregnancies have been recognized. However, the decision of whether to reduce to twins or a singleton is still difficult. Some previous small size studies compared triplets reduced to twins and to singletons as follows. Haas et al. compared 55 twins and 19 singletons reduced from triplets and showed that reduction to a singleton resulted in a longer GA at delivery and higher birth weight (21). However, some researchers still believe that MFPR from triplets to singletons is associated with a higher risk of pregnancy loss (22). In our study, triplets reduced to singletons did not increase pregnancy loss at <24 weeks compared with triplets reduced to twins. Triplets reduced to singletons had better outcomes in almost every aspect compared with primary twins, including a longer GA, lower preterm delivery rate, lower cesarean section rate, higher birth weight, and lower frequency of LBW or SGA newborns.

For twin pregnancies, there is still controversy regarding whether to perform MFPR. A previous study (23) showed that in the twins reduced to singletons group, the percentage of women without any surviving child was significantly higher compared with the ongoing twin. Gupta et al. (24) reported that reduction of twin pregnancies decreased the risk of preterm delivery at <37 weeks and birth weight below the 10th percentile, but not the risk of preterm birth at <34 weeks or birth weight below the 5th percentile. There is no doubt that an increased risk of adverse pregnancy outcomes is associated with twin pregnancies (3, 4, 25). In our study, we found that twins reduced to singletons had better outcomes in almost every aspect compared with primary twins, including a longer GA, lower preterm delivery rate, lower cesarean section rate, higher birth weight, and lower frequency of LBW or SGA newborns. Importantly, twins reduced to singletons did not increase pregnancy loss at <24 weeks. These findings are consistent with previous studies (26) (27), which suggest that MFPR from twins to singletons has a clear advantage for twin pregnancies.

The conclusion can be drawn from previous studies and the present study that MFPR improves the outcomes of triplet or twin pregnancies. However, there is still controversy whether reduced singletons or twins after MFPR have the same pregnancy outcomes as non–reduced singletons or twins.

To date, the findings of studies have been inconsistent with the pregnancy outcomes of reduced twins and primary twins. In some studies, reduced twins have similar outcomes compared with primary twins. Hershko–Klement et al. (28) evaluated the pregnancy outcomes of 70 twins after reduction, and found that the mean GA at delivery and birth weight were comparable between the reduced and non–reduced twins. Lipitz et al. (29) also showed that the mean GA at delivery was similar in reduced and non–reduced twins, as well as the risk of LBW. Our study included 331 women with twin pregnancies who underwent MFPR. This is the largest cohort described to date and it provides a more precise estimation of preterm delivery and birth weight. In the current study, most of the pregnancy outcomes were comparable between twins reduced from triplets and primary twins, including the rates of preterm delivery at <32, <34, and <37 weeks, pregnancy loss at <24 weeks, abortion of one fetus, and cesarean section. However, the probability for women who had twins reduced from triplets to have a LBW or SGA neonate was higher than that for those who had primary twins. The findings of our study are consistent with those presented by Cheang et al. (30) and Hwang et al. (16), which suggested the higher risk of prematurity in reduced twins.

Due to the sample size, we combined the triplets reduced to singletons group and twins reduced to singletons group for statistics analyze. Triplets/twins reduced to singletons were more likely to have preterm delivery at <32, <34, and <37 weeks. Birth weight of reduced singletons was 285g lighter than that of primary singletons. Women who had triplets/twins reduced to singletons were more likely to have a LBW, VLBW or SGA neonate compared with women who had primary singletons. Moreover, the rates of pregnancy loss at <24 weeks and cesarean section were comparable between the two groups in our study. Consistent with our study, van de Mheen al (23). found that reduced singletons had a shorter GA at delivery and lower birth weight than primary singletons.

The major strength of this study is that it is the largest study to analyze the pregnancy outcomes of twin or triplet pregnancies undergoing MFPR to date. In this single–center study, all of the experienced operators followed a unified operating standard, reducing the interference caused by operating variability. Moreover, we included patients over a long timeframe, which increased the validity of the study. However, this study has some limitations. Although this is the largest study to date, the numbers of some subgroups were small, which might have restricted our ability to detect differences in some pregnancy outcomes of low probability, such as extreme preterm delivery and VLBW. Data regarding pregnancy complications and perinatal mortality were not available. Our study has a large sample size over a long period. Over this time, the outcomes of IVF/ICSI pregnancies in our center were relatively stable, and all MFPR procedures were performed by the same five highly skilled physicians, thus ensuring the reliability of this study. Thus, the year of conception or birth was not put in regression model in our study, sine time-changes might not significantly contribute to apparent group differences. In addition, some baseline characteristics were different between groups, because this was not a randomized trial owing to the fact that randomization of patients was not applicable. To reduce interference of confounding factors, we used multiple regressions to verify our results.

In conclusion, MFPR is a relatively safe and efficacious procedure based on our findings, but the objective of our study was not to advocate MFPR. MFPR could improve but still cannot completely reverse adverse pregnancy outcomes of multiple pregnancies. The best way to prevent multiple pregnancies and all related risks is limiting the number of transferred embryos and the advocating of SET. For those infertile couples seeking for ART, we must attach particular importance to inform them the risk of multiple pregnancies and benefits of SET. We should be aware that it is SET, not MFPR, the optimal choice for reducing the risk of multiple pregnancies from the beginning (31). All of this information should be considered when counselling couples about the number of embryos transferred or women with multiple pregnancies who are considering MFPR. The long-term impact of MFPR on the health of the offspring should also been further investigated in the future.

## Data Availability Statement

The raw data supporting the conclusions of this article will be made available by the authors, without undue reservation.

## Ethics Statement

The studies involving human participants were reviewed and approved by the Women’s hospital, Zhejiang University School of Medicine. The patients/participants provided their written informed consent to participate in this study.

## Author Contributions

ZY is the principal investigator for the study. ZY and TM conceived the present study and carried out most of the research for this study. All authors contributed to the analysis and interpretation of the data. TM interpreted the results and wrote the manuscript, which was critically revised by all authors. All the authors approved the submitted version.

## Funding

This work was supported by grants from National Key R&D Program of China (2021YFC2700603), the Zhejiang Natural Science Foundation of China (NO. LQ22H040006 and Q22H049995), the National Natural Science Foundation of China (No. 82101759 and NO.81803245), the Program for Key Subjects of Zhejiang Province in Medicine & Hygiene.

## Conflict of Interest

The authors declare that the research was conducted in the absence of any commercial or financial relationships that could be construed as a potential conflict of interest.

## Publisher’s Note

All claims expressed in this article are solely those of the authors and do not necessarily represent those of their affiliated organizations, or those of the publisher, the editors and the reviewers. Any product that may be evaluated in this article, or claim that may be made by its manufacturer, is not guaranteed or endorsed by the publisher.

## Supplementary Material

Supplementary Table 1 Pregnancy outcomes: triplets or twins reduced to singletons versus primary singletons.

Supplementary Figure 1 The birth weight of newborns in different groups.
