# Supplementary material for: Fetal Reduction Could Improve but Not Completely Reverse the Pregnancy Outcomes of Multiple Pregnancies: Experience From a Single Center
Source: Front Endocrinol (Lausanne). 2022 Jun 24;13:851167. doi: 10.3389/fendo.2022.851167 (PMC9263074; doi:10.3389/fendo.2022.851167)
Supplement: Supplementary Table 1 — Pregnancy outcomes: triplets or twins reduced to singletons versus primary singletons. [file Table_1.docx]

**Supplemental Table 1**

**Pregnancy outcomes: triplets or twins reduced to singletons versus primary singletons**

|  | **Primary**  **singletons**  **(N=6853)** | **Singletons reduced from twins**  **(N=126)** | **Triplets reduced**  **to singletons**  **(N=45)** | **Unadjusted**  ***P***  **Value 1** | **Unadjusted**  **OR1**  **(95%CI)** | **Unadjusted**  ***P* Value 2** | **Unadjusted**  **OR2**  **(95%CI)** | **Adjusted**  ***P* Value 1** | **Adjusted OR1**  **(95%CI)** | **Adjusted**  ***P* Value 2** | **Adjusted OR2**  **(95%CI)** |
| --- | --- | --- | --- | --- | --- | --- | --- | --- | --- | --- | --- |
| **GA at delivery** | 39.00  (38.00-40.00) | 38.79  (37.46-39.43) | 39.07  (38.25-40.04) | 0.203 | - | 0.082 | - | 0.046 | - | 0.575 | - |
| **Delivery<32weeks** | 65(0.9) | 5(4.0) | 1(2.2) | 0.004 | 4.32  (1.71-10.91) | 0.915 | 2.37  (0.32-17.49) | 0.003 | 1.27  (1.09-1.48) | 0.350 | 1.17 (0.84-1.64) |
| **Delivery<34weeks** | 119(1.7) | 7(5.6) | 2(4.4) | 0.004 | 3.33  (1.52-7.29) | 0.418 | 2.63  (0.63-10.99) | 0.003 | 1.23  (1.07-1.40) | 0.124 | 1.21 (0.95-1.53) |
| **Delivery<37weeks** | 500(7.3) | 21(16.7) | 6(13.3) | <0.001 | 2.54  (1.58-4.10) | 0.207 | 1.96  (0.82-4.64) | <0.001 | 1.16  (1.07-1.26) | 0.105 | 1.13 (0.98-1.30) |
| **Pregnancy loss <24weeks** | 371(5.4) | 6(4.8) | 3(6.7) | 0.748 | 0.87  (0.38-2.00) | 0.968 | 1.25  (0.39-4.05) | 0.503 | 0.95  (0.83-1.10) | 0.754 | 1.03 (0.85-1.26) |
| **Live birth** | 6450 (94.1) | 120 (95.2) | 42 (93.3) | 0.596 | 1.25 (0.55-2.86） | 0.823 | 0.88 (0.27-2.83) | 0.380 | 1.06 (0.93-1.22) | 0.883 | 0.99 (0.81-1.20) |
| **Caesarean section** | 4871/6463  (75.4) | 92/120  (76.7) | 28/42  (66.7) | 0.743 | 1.07  (0.70-1.65) | 0.192 | 0.65  (0.34-1.25) | 0.742 | 0.99  (0.92-1.06) | 0.178 | 0.92 (0.83-1.04) |
| **Birth weight (g)** | 3340  (3038.73-3650) | 3080  (2750-3350) | 3050  (2775-3300) | <0.001 | - | <0.001 | - | <0.001 | - | <0.001 | - |
| **LBW** | 295(4.3) | 17(13.5) | 4(8.9) | <0.001 | 3.47  (2.05-5.86) | 0.255 | 2.17  (0.78-6.10) | <0.001 | 1.22  (1.12-1.34) | 0.135 | 1.14 (0.96-1.36) |
| **VLBW** | 29(0.4) | 4(3.2) | 0 | 0.003 | 7.72  (2.67-22.28) | 1.000 | - | <0.001 | 1.39 (1.16-1.66) | 0.998 | - |
| **SGA** | 451(6.6) | 17(13.5) | 8(17.8) | 0.002 | 2.21  (1.32-3.72) | 0.003 | 3.07  (1.42-6.63) | 0.001 | 1.15  (1.06-1.26) | 0.006 | 1.20 (1.06-1.37) |

Data are presented as median (IQR) or number (%). Mann–Whitney U tests and Chi-square tests or Fisher’s exact test were used for unadjusted analysis. Logistic regression and linear regression were used for adjusting certain confounders, including maternal age at conception, maternal BMI before pregnancy, type of infertility, ART methods, embryo transplantation, source of semen. *P*1 represented for the differences between twins reduced to singletons and primary singletons; *P*2 represented for the differences between triplets reduced to singletons and primary singletons;

Abbreviations: GA, gestational age; LBW, low birth weight; VLBW, very low birth weight; SGA, Small for gestational age; OR, odds ratio; CI, confidence interval.
